# Verifying Quantum Communication Protocols with Ground Bisimulation

**DOI:** 10.1007/978-3-030-45237-7_2

**Published:** 2020-03-13

**Authors:** Xudong Qin, Yuxin Deng, Wenjie Du

**Affiliations:** 8 Johannes Kepler University, Linz, Austria; 9 University of Birmingham, Birmingham, UK; 10 Shanghai Key Laboratory of Trustworthy Computing, MOE International Joint Lab of Trustworthy Software, and International Research Center of Trustworthy Software, East China Normal University, Shanghai, China; 11 Peng Cheng Laboratory, Shenzhen, China; 12 Shanghai Normal University, Shanghai, China

**Keywords:** Quantum process algebra, Bisimulation, Verification, Quantum communication protocols

## Abstract

One important application of quantum process algebras is to formally verify quantum communication protocols. With a suitable notion of behavioural equivalence and a decision method, one can determine if an implementation of a protocol is consistent with its specification. Ground bisimulation is a convenient behavioural equivalence for quantum processes because of its associated coinduction proof technique. We exploit this technique to design and implement two on-the-fly algorithms for the strong and weak versions of ground bisimulation to check if two given processes in quantum CCS are equivalent. We then develop a tool that can verify interesting quantum protocols such as the BB84 quantum key distribution scheme.

### 1 Introduction

Process algebras provide a useful formal method for specifying and verifying concurrent systems. Their extensions to the quantum setting have also appeared in the literature. For example, Jorrand and Lalire [18,21] defined the *Quantum Process Algebra* (QPAlg) and presented a branching bisimulation to identify quantum processes with the same branching structure. Gay and Nagarajan [15] developed *Communicating Quantum Processes* (CQP), for which Davidson [6] established a bisimulation congruence. Feng et al. [10] have proposed a quantum variant of Milner's CCS [23], called qCCS, and a notion of probabilistic bisimulation for quantum processes, which is then improved to be a general notion of bisimulation that enjoys a congruence property [12]. Later on, motivated by [25], Deng and Feng [9] defined an open bisimulation for quantum processes

* Supported by the National Natural Science Foundation of China (61672229, 61832015) and the Inria-CAS joint project Quasar.

that makes it possible to separate ground bisimulation and the closedness under super-operator applications, thus providing not only a neater and simpler definition, but also a new technique for proving bisimilarity. In order to avoid the problem of instantiating quantum variables by potentially infinitely many quantum states, Feng et al. [11] extended the idea of symbolic bisimulation [17] for value-passing CCS and provided a symbolic version of open bisimulation for qCCS. They proposed an algorithm for checking symbolic ground bisimulation.

In the current work, we consider the ground bisimulation proposed in [9]. We put forward an on-the-fly algorithm to check if two given processes in qCCS with fixed initial quantum states are ground bisimilar. The algorithm is simpler than the one in [11] because the initial quantum states are determined for the former but can be parametric for the latter. Moreover, in many applications, we are only interested in the correctness of a quantum protocol with a predetermined input of quantum states. This is especially the case in the design stage of a protocol or in the debugging of a program.

The ground bisimulation defined in [9] is a notion of weak bisimulation because a strong transition can be matched by a weak transition where invisible actions are abstracted away. We also consider a strong version where all actions are visible, for which we have a simpler algorithm. Both algorithms are obtained by adapting the on-the-fly algorithm for checking probabilistic bisimulations [8,7], which in turn has its root in similar algorithms for checking classical bisimulations [14,17]. The basic idea is as follows. A quantum process with an initial quantum state forms a configuration. We describe the operational behaviour of a configuration as a probabilistic labelled transition system (pLTS), where probabilistic transitions arise naturally because measuring a quantum system can entail a probability distribution of post-measurement quantum systems. Ground bisimulations are a strengthening of probabilistic bisimulations by imposing some constraints on quantum variables and the environment states of processes. The skeleton of the algorithm for the strong ground bisimulation resembles to that for strong probabilistic bisimulation [8]. The algorithm for the (weak) ground bisimulation is inspired by [28] and uses as a subroutine a procedure in the aforementioned work. The procedure reduces the problem of finding a matching weak transition to a linear programming problem that can be solved in polynomial time. We have developed a tool that implements both algorithms and can check if two given configurations are strongly or weakly bisimilar. It is useful to validate whether an implementation of a protocol is equivalent to the specification. We have conducted experiments on a few interesting quantum protocols including super-dense coding, teleportation, secret sharing, and several quantum key distribution protocols, in particular the BB84 protocol [5], to analyse the functional correctness of the protocols.

*Other related work* Ardeshir-Larijani et al. [3] proposed a quantum variant of CCS to describe quantum protocols. The syntax of that variant is similar to qCCS but its semantics is very different. The behaviour of a concurrent process is a finite tree and an interleaving is a path from the root to a leaf. By interpreting an interleaving as a superoperator [26], the semantics of a process is a set of superoperators. The equivalence checking between two processes boils down to the equivalence checking between superoperators, which is accomplished by using the stabiliser simulation algorithm invented by Aaronson and Gottesman [1]. Ardeshir-Larijani et al. have implemented their approach in an equivalence checker in Java and verified several quantum protocols from teleportation to secret sharing. However, they are not able to handle the BB84 quantum key distribution protocol because its correctness cannot be specified as an equivalence between interleavings. Our approach is based on ground bisimulation and keeps all the branching behaviour of a concurrent process. Our algorithms for checking ground bisimulations are influenced by the on-the-fly algorithm of Hennessy and Lin for value-passing CCS [17]. We are inspired by the probabilistic bisimulation checking algorithm of Baier et al. [4] for the strong version of ground bisimulation, and by the weak bisimulation checking algorithm of Turrini and Hermanns [28] for the weak version.

Kubota et al. [20] implemented a semi-automated tool to check a notion of symbolic bisimulation and used it to verify the equivalence of BB84 and another quantum key distribution protocol based on entanglement distillation [27]. There are two main differences between their work and ours. (1) Their tool is based on equational reasoning and thus requires a user to provide equations while our tool is fully automatic. (2) Their semantic interpretation of measurement is different and entails a kind of linear-time semantics for quantum processes that ignores the timepoints of the occurrences of probabilistic branches. However, we use a branching-time semantics. For instance, the occurrence of a measurement before or after a visible action is significant for our semantics but not for the semantics proposed in [20].

Besides equivalence checking, based on either superoperators or bisimulations as mentioned above, model checking is another feasible approach to verify quantum protocols. For instance, Gay et al. developed the QMC model checker [16]. Feng et al. implemented the tool QPMC [13] to model check quantum programs and protocols. There are also other approaches for verifying quantum systems. Abramsky and Coecke [2] proposed a categorical semantics for quantum protocols. Quantomatic [19] is a semi-automated tool based on graph rewriting. Ying [30] established a quantum Hoare logic, which has been implemented in a theorem prover [22].

The rest of the paper is structured as follows. In Section 2 we recall the syntax and semantics of the quantum process algebra qCCS. In Section 3 we present an algorithm for checking ground bisimulations. In Section 4 we report the implementation of the algorithm and some experimental results on verifying a few quantum communication protocols. Finally, we conclude in Section 5 and discuss some future work.

### 2 Quantum CCS

We introduce a quantum extension of classical CCS (qCCS) which was originally studied in [10,29,12]. Three types of data are considered in qCCS: as classical

$$\begin{array}{rlrl} qv(\textbf{nil}) &= \emptyset & qv(\tau.P) &= qv(P) \\ qv(c?x.P) &= qv(P) & qv(c!e.P) &= qv(P) \\ qv(\underline{c}?q.P) &= qv(P) - \{q\} & qv(\underline{c}!q.P) &= qv(P) \cup \{q\} \\ qv(\mathcal{E}[\tilde{q}].P) &= qv(P) \cup \tilde{q} & qv(M[\tilde{q};x].P) &= qv(P) \cup \tilde{q} \\ qv(P+Q) &= qv(P) \cup qv(Q) & qv(P \mid\mid Q) &= qv(P) \cup qv(Q) \\ qv(P[f]) &= qv(P) & qv(P \backslash L) &= qv(P) \\ qv(\textbf{if } b \textbf{ then } P) &= qv(P) & qv(A(\tilde{q};\tilde{x})) &= \tilde{q}. \end{array}$$

**Fig. 1.** Free quantum variables data we have `Bool` for booleans and `Real` for real numbers, and as quantum data we have `Qbt` for qubits. Consequently, two countably infinite sets of variables are assumed: $cVar$ for classical variables, ranged over by $x, y, ...$, and $qVar$ for quantum variables, ranged over by $q, r, ....$ We assume a set $Exp$, which includes $cVar$ as a subset and is ranged over by $e, e', \ldots$, of classical data expressions over `Real`, and a set of boolean-valued expressions $BExp$, ranged over by $b, b', \ldots$, with the usual boolean constants `true`, `false`, and operators $\neg$, $\wedge$, $\vee$, and $\rightarrow$. In particular, we let $e \bowtie e'$ be a boolean expression for any $e, e' \in Exp$ and $\bowtie \in \{>, <, \geq, \leq, =\}$. We further assume that only classical variables can occur freely in both data expressions and boolean expressions. Two types of channels are used: $cChan$ for classical channels, ranged over by $c, d, ...$, and $qChan$ for quantum channels, ranged over by $\underline{c}, \underline{d}, ....$ A relabelling function $f$ is a map on $cChan \cup qChan$ such that $f(cChan) \subseteq cChan$ and $f(qChan) \subseteq qChan$. Sometimes we abbreviate a sequence of distinct variables $q_1, ..., q_n$ into $\tilde{q}$.

The terms in qCCS are given by:

$$\begin{aligned} P, Q ::= \textbf{nil} \mid \tau.P \mid c?x.P \mid c!e.P \mid \underline{c}?q.P \mid \underline{c}!q.P \mid \mathcal{E}[\tilde{q}].P \mid M[\tilde{q};x].P \mid \\ P+Q \mid P \mid\mid Q \mid P[f] \mid P \backslash L \mid \textbf{if } b \textbf{ then } P \mid A(\tilde{q};\tilde{x}) \end{aligned}$$

where $f$ is a relabelling function and $L \subseteq cChan \cup qChan$ is a set of channels. Most of the constructors are standard as in CCS [23]. We briefly explain a few new constructors. The process $\underline{c}?q.P$ receives a quantum datum along quantum channel $\underline{c}$ and evolves into $P$, while $\underline{c}!q.P$ sends out a quantum datum along quantum channel $\underline{c}$ before evolving into $P$. The symbol $\mathcal{E}$ represents a trace-preserving super-operator applied on the quantum system referred to by the variables $\tilde{q}$. The process $M[\widetilde{q};x].P$ measures the state of qubits $\widetilde{q}$ according to the observable $M$ and stores the measurement outcome into the classical variable $x$ of $P$.

Free classical variables can be defined in the usual way, except for the fact that the variable $x$ in the quantum measurement $M[\tilde{q};x]$ is bound. A process $P$ is closed if it contains no free classical variable, i.e. $fv(P) = \emptyset$.

The set of free quantum variables for process $P$, denoted by $qv(P)$ can be inductively defined as in Figure 1. For a process to be legal, we require that

1. $q \notin qv(P)$ in the process $\underline{c}!q.P$;
2. $qv(P) \cap qv(Q) = \emptyset$ in the process $P \mid\mid Q$;

*(Tau)*
$$\langle \tau.P,\rho\rangle \xrightarrow{\tau} \langle P,\rho\rangle$$

*(C-Inp)*
$$\frac{v \in \texttt{Real}}{\langle c?x.P,\rho\rangle \xrightarrow{c?v} \langle P[v/x],\rho\rangle}$$

*(C-Outp)*
$$\frac{v = [\![e]\!]}{\langle c!e.P,\rho\rangle \xrightarrow{c!v} \langle P,\rho\rangle}$$

*(C-Com)*
$$\frac{\langle P_1,\rho\rangle \xrightarrow{c?v} \langle P_1',\rho\rangle \qquad \langle P_2,\rho\rangle \xrightarrow{c!v} \langle P_2',\rho\rangle}{\langle P_1 \,||\, P_2,\rho\rangle \xrightarrow{\tau} \langle P_1' \,||\, P_2',\rho\rangle}$$

*(Q-inp)*
$$\frac{r \notin qv(\underline{c}?q.P)}{\langle \underline{c}?q.P,\rho\rangle \xrightarrow{\underline{c}?r} \langle P[r/q],\rho\rangle}$$

*(Q-Outp)*
$$\langle \underline{c}!q.P,\rho\rangle \xrightarrow{\underline{c}!q} \langle P,\rho\rangle$$

*(Q-Com)*
$$\frac{\langle P_1,\rho\rangle \xrightarrow{\underline{c}?r} \langle P_1',\rho\rangle \qquad \langle P_2,\rho\rangle \xrightarrow{\underline{c}!r} \langle P_2',\rho\rangle}{\langle P_1 \,||\, P_2,\rho\rangle \xrightarrow{\tau} \langle P_1' \,||\, P_2',\rho\rangle}$$

*(Oper)*
$$\langle \mathcal{E}[\tilde{q}].P,\rho\rangle \xrightarrow{\tau} \langle P,\mathcal{E}_{\tilde{q}}(\rho)\rangle$$

*(Meas)*
$$\frac{M = \sum_{i\in I} \lambda_i E^i \qquad p_i = tr(E^i_{\tilde{q}}\rho)}{\langle M[\tilde{q};x].P,\rho\rangle \xrightarrow{\tau} \sum_{i\in I} p_i \langle P[\lambda_i/x], E^i_{\tilde{q}}\rho E^i_{\tilde{q}}/p_i\rangle}$$

*(Int)*
$$\frac{\langle P_1,\rho\rangle \xrightarrow{\alpha} \Delta \qquad qbv(\alpha)\cap qv(P_2)=\emptyset}{\langle P_1 \,||\, P_2,\rho\rangle \xrightarrow{\alpha} \Delta \,||\, P_2}$$

*(Sum)*
$$\frac{\langle P_1,\rho\rangle \xrightarrow{\alpha} \Delta}{\langle P_1 + P_2,\rho\rangle \xrightarrow{\alpha} \Delta}$$

*(Rel)*
$$\frac{\langle P,\rho\rangle \xrightarrow{\alpha} \Delta}{\langle P[f],\rho\rangle \xrightarrow{f(\alpha)} \Delta[f]}$$

*(Res)*
$$\frac{\langle P,\rho\rangle \xrightarrow{\alpha} \Delta \qquad cn(\alpha)\cap L=\emptyset}{\langle P\backslash L,\rho\rangle \xrightarrow{\alpha} \Delta\backslash L}$$

*(Cho)*
$$\frac{\langle P,\rho\rangle \xrightarrow{\alpha} \Delta \qquad [\![b]\!] = \texttt{true}}{\langle \textbf{if } b \textbf{ then } P,\rho\rangle \xrightarrow{\alpha} \Delta}$$

*(Cons)*
$$\frac{\langle P[\tilde{v}/\tilde{x},\tilde{r}/\tilde{q}],\rho\rangle \xrightarrow{\alpha} \Delta \qquad A(\tilde{x},\tilde{q}) := P}{\langle A(\tilde{v},\tilde{r}),\rho\rangle \xrightarrow{\alpha} \Delta}$$

**Fig. 2.** Operational semantics of qCCS. Here in rule *(C-Outp)*, $[\![e]\!]$ is the evaluation of $e$, and in rule *(Meas)*, $E^i_{\tilde{q}}$ denotes the operator $E^i$ acting on the quantum systems $\tilde{q}$.

3. Each constant $A(\tilde{q};\tilde{x})$ has a defining equation $A(\tilde{q};\tilde{x}) := P$, where $P$ is a term with $qv(P) \subseteq \tilde{q}$ and $fv(P) \subseteq \tilde{x}$.

The first condition says that a quantum system will not be referenced after it has been sent out. This is a requirement of the quantum no-cloning theorem. The second condition says that parallel composition $||$ models separate parties that never reference a quantum system simultaneously.

Throughout the paper we implicitly assume the convention that processes are identified up to $\alpha$-conversion, bound variables differ from each other and they are different from free variables.

Before introducing the operational semantics of qCCS processes, we review the model of probabilistic labelled transition systems (pLTSs). Later on we will interpret the behaviour of quantum processes in terms of pLTSs because quantum measurements give rise to probability distributions naturally.

We begin with some notations. A (discrete) probability distribution over a set $S$ is a function $\Delta\colon S \to [0,1]$ with $\sum_{s\in S}\Delta(s)=1$; the support of such a $\Delta$ is the set $\lceil\Delta\rceil = \{\, s \in S \mid \Delta(s) > 0\,\}$. The point distribution $\overline{s}$ assigns probability 1 to $s$ and 0 to all other elements of $S$, so that $\lceil\overline{s}\rceil = \{s\}$. We only need to use distributions with finite supports, and let $\mathit{Dist}(S)$ denote the set of finite support distributions over $S$, ranged over by $\Delta$, $\Theta$, etc. If $\sum_{k\in K} p_k = 1$ for some collection of $p_k \geq 0$, and the $\Delta_k$ are distributions, then so is $\sum_{k\in K} p_k \cdot \Delta_k$ with $(\sum_{k\in K} p_k \cdot \Delta_k)(s) \;=\; \sum_{k\in K} p_k \cdot \Delta_k(s)$.

**Definition 1.** *A* probabilistic labelled transition system *is a triple* $\langle S, \mathsf{Act}_\tau, \rightarrow\rangle$*, where $S$ is a set of states,* $\mathsf{Act}_\tau$ *is a set of visible actions* $\mathsf{Act}$ *augmented with the invisible action $\tau$, and* $\rightarrow\, \subseteq S \times \mathsf{Act}_\tau \times \mathit{Dist}(S)$ *is the transition relation.*

We often write $s \xrightarrow{\alpha} \Delta$ for $(s,\alpha,\Delta) \in \rightarrow$. In pLTSs we not only consider relations between states, but also relations between distributions. Therefore, we make use of the lifting operation below [7].

**Definition 2.** *Let $\mathcal{R} \subseteq S \times S$ be a relation between states. Then $\mathcal{R}^\circ \subseteq \mathit{Dist}(S) \times \mathit{Dist}(S)$ is the smallest relation that satisfies the two rules: (i) $s\ \mathcal{R}\ s'$ implies $\overline{s}\ \mathcal{R}^\circ\ \overline{s'}$; (ii) $\Delta_i\ \mathcal{R}^\circ\ \Theta_i$ for all $i \in I$ implies $(\sum_{i\in I} p_i \cdot \Delta_i)\ \mathcal{R}^\circ\ (\sum_{i\in I} p_i \cdot \Theta_i)$ for any $p_i \in [0,1]$ with $\sum_{i\in I} p_i = 1$, where $I$ is a finite index set.*

We apply this operation to the relations $\xrightarrow{\alpha}$ in the pLTS for $\alpha \in \mathsf{Act}_\tau$, where we also write $\xrightarrow{\alpha}$ for $(\xrightarrow{\alpha})^\circ$. Thus as source of a relation $\xrightarrow{\alpha}$ we now also allow distributions. But note that $\overline{s} \xrightarrow{\alpha} \Delta$ is more general than $s \xrightarrow{\alpha} \Delta$ because if $\overline{s} \xrightarrow{\alpha} \Delta$ then there is a collection of distributions $\Delta_i$ and probabilities $p_i$ such that $s \xrightarrow{\alpha} \Delta_i$ for each $i \in I$ and $\Delta = \sum_{i\in I} p_i \cdot \Delta_i$ with $\sum_{i\in I} p_i = 1$.

We write $s \xrightarrow{\hat{\tau}} \Delta$ if either $s \xrightarrow{\tau} \Delta$ or $\Delta = \overline{s}$. We define weak transitions $\stackrel{\hat{a}}{\Longrightarrow}$ by letting $\stackrel{\hat{\tau}}{\Longrightarrow}$ be the reflexive and transitive closure of $\xrightarrow{\hat{\tau}}$ and writing $\Delta \stackrel{\hat{a}}{\Longrightarrow} \Theta$ for $a \in \mathsf{Act}$ whenever $\Delta \stackrel{\hat{\tau}}{\Longrightarrow}\xrightarrow{a}\stackrel{\hat{\tau}}{\Longrightarrow} \Theta$. If $\Delta = \overline{s}$ is a point distribution, we often write $s \stackrel{\hat{a}}{\Longrightarrow} \Theta$ instead of $\overline{s} \stackrel{\hat{a}}{\Longrightarrow} \Theta$.

We now give the semantics of qCCS. For each quantum variable $q$ we assume a 2-dimensional Hilbert space $\mathcal{H}_q$. For any nonempty subset $S \subseteq \mathit{qVar}$ we write $\mathcal{H}_S$ for the tensor product space $\bigotimes_{q\in S} \mathcal{H}_q$ and $\mathcal{H}_{\overline{S}}$ for $\bigotimes_{q\notin S} \mathcal{H}_q$. In particular, $\mathcal{H} = \mathcal{H}_{\mathit{qVar}}$ is the state space of the whole environment consisting of all the quantum variables, which is a countably infinite dimensional Hilbert space.

Let $P$ be a closed quantum process and $\rho$ a density operator on $\mathcal{H}$[1], the pair $\langle P, \rho\rangle$ is called a *configuration*. We write $\mathit{Con}$ for the set of all configurations, ranged over by $\mathcal{C}$ and $\mathcal{D}$. We interpret qCCS with a pLTS whose states are all the configurations definable in the language, and whose transitions are determined by the rules in Figure 2; we have omitted the obvious symmetric counterparts to the rules *(C-Com)*, *(Q-Com)*, *(Int)* and *(Sum)*. The set of actions $\mathsf{Act}$ takes the following form, consisting of classical/quantum input/output actions.

$$\mathsf{Act} = \{c?v, c!v \mid c \in \mathit{cChan}, v \in \mathtt{Real}\} \cup \{\underline{c}?r, \underline{c}!r \mid \underline{c} \in \mathit{qChan}, r \in \mathit{qVar}\}$$

[1] As $\mathcal{H}$ is infinite dimensional, $\rho$ should be understood as a density operator on some finite dimensional subspace of $\mathcal{H}$ which contains $\mathcal{H}_{qv(P)}$.

We use $cn(\alpha)$ for the set of channel names in action $\alpha$. For example, we have $cn(\underline{c}?x) = \{\underline{c}\}$ and $cn(\tau) = \emptyset$.

In the first eight rules in Figure 2, the targets of arrows are point distributions, and we use the slightly abbreviated form $\mathcal{C} \xrightarrow{\alpha} \mathcal{C}'$ to mean $\mathcal{C} \xrightarrow{\alpha} \overline{\mathcal{C}'}$.

The rules use the obvious extension of the function $||$ on terms to configurations and distributions. To be precise, $\mathcal{C} \mid\mid P$ is the configuration $\langle Q \mid\mid P, \rho \rangle$ where $\mathcal{C} = \langle Q, \rho \rangle$, and $\Delta \mid\mid P$ is the distribution defined by:

$$(\Delta \mid\mid P)(\langle Q, \rho \rangle) \stackrel{def}{=} \begin{cases} \Delta(\langle Q', \rho \rangle) & \text{if } Q = Q' \mid\mid P \text{ for some } Q' \\ 0 & \text{otherwise.} \end{cases}$$

Similar extension applies to $\Delta[f]$ and $\Delta \backslash L$.

Suppose there is a configuration $\mathcal{C} = \langle P, \rho \rangle$, the partial trace over system $P$ at such state can be defined as $tr_{qv(P)}(\rho)$ whose result is a reduced density operator representing the state of the environment. We give the definition of ground bisimulation and bisimilarity as follows.

**Definition 3 ([9]).** *A relation $\mathcal{R} \subseteq Con \times Con$ is a* ground simulation *if for any $\mathcal{C} = \langle P, \rho \rangle$, $\mathcal{D} = \langle Q, \sigma \rangle$, $\mathcal{C} \ \mathcal{R} \ \mathcal{D}$ implies that $qv(P) = qv(Q)$, $tr_{qv(P)}(\rho) = tr_{qv(Q)}(\sigma)$, and*

– *whenever $\mathcal{C} \xrightarrow{\alpha} \Delta$, there is some distribution $\Theta$ with $\mathcal{D} \stackrel{\hat{\alpha}}{\Longrightarrow} \Theta$ and $\Delta \ \mathcal{R}^\circ \ \Theta$.*

*A relation $\mathcal{R}$ is a* ground bisimulation *if both $\mathcal{R}$ and $\mathcal{R}^{-1}$ are ground simulations. We denote by $\approx$ the largest ground bisimulation, called* ground bisimilarity*. If the above weak transition $\mathcal{D} \stackrel{\hat{\alpha}}{\Longrightarrow} \Theta$ is replaced by a strong transition $\mathcal{D} \xrightarrow{\alpha} \Theta$, we obtain a* strong ground bisimulation*.*

In the rest of the paper, we mainly focus on ground bisimulation and only briefly mention the algorithm for checking strong ground bisimulation.

### 3 Algorithm

We present an on-the-fly algorithm to check if two configurations are ground bisimilar.

The algorithm maintains two sets $NonBisim$ and $Bisim$ to keep non-bisimilar and bisimilar state pairs, respectively. When the algorithm terminates, $Bisim$ should contain all the state pairs satisfying the bisimulation relation.

The function **Bisim**$(t, u)$, as shown in Algorithm 1, is the main function of the algorithm, which attempts to find the smallest bisimulation containing the pair $(t, u)$. It initialises $Bisim$ and a set named $Visited$ to store the visited pairs, then calls the function **Match** to search for a bisimulation. The function **Match**$(t, u, Visited)$ invokes a depth-first traversal to match a pair of states $(t, u)$ with all their possible behaviours. The set $Visited$ is updated before the traversal for detecting loops. We also match the behaviours of $t$ and $u$ from both directions as we are checking bisimulations. Two states are deemed non-bisimilar in three cases:

- one state has a transition that cannot be matched by any possible weak transition from the other;
- they do not have the same set of free quantum variables;
- the density operators of them corresponding to their quantum registers are different.

The first case is checked by **MatchAction**, and the other two are done in **Match**. We add a pair of states to $NonBisim$ if one of the three cases above has occurred. Otherwise, it will be stored in $Bisim$.

An auxiliary function **Act**$(t)$ is invoked in **Match** to discover the next action that $t$ can perform. If $t$ have no more action to perform the function will return an empty set.

The function **MatchAction**$(\alpha, t, u, Visited)$ checks the equivalence of configurations through comparing their transitions. The function recursively discovers the next equivalent state pairs between the target states of the transitions. Technically, it checks the condition that if $t \xrightarrow{\alpha} \Delta$ then there exists some $\Theta$ such that $u \stackrel{\hat{\alpha}}{\Longrightarrow} \Theta$ and $\Delta \;\; \mathcal{R}^\circ \;\; \Theta$. Here we use as a subroutine a procedure of [28] to reduce the problem to a linear programming problem that can be solved in polynomial time. The problem is defined in Appendix. In **MatchAction**, we introduce a predicate **LP**$(\Delta, u, \alpha, \mathcal{R})$ which is true if and only if the linear programming problem has a solution. We invoke the function **Close** to construct an equivalence relation $\mathcal{R}$ between $S$ and the states in the support of the target distribution. Note that in Lines 28 and 34 we have two distinct cases because in output actions the emitted values are required to be equal, which are unlike other types of actions.

In general, there are loops in pLTSs. When a state pair to be considered is already contained in $Visited$ it will be assumed to be bisimilar and added to $Assumed$ (Lines 42-43). Later on, if the pair of states are found to be nonbisimilar, the pair will be added to $NonBisim$ and a wrong assumption exception (Lines 18-21) will be raised to restart the checking process from the original pair of states. Then **Bisim**$(t, u)$ renews the sets $Bisim$, $Visited$ and $Assumed$ to remove the pairs checked under the wrong assumption (Lines 4-6).

**Algorithm 1** Checking ground bisimulation

**Require:** Two pLTSs with initial configurations $t$ and $u$.
**Ensure:** A boolean value $b_{res}$ indicating if the two pLTSs are ground bisimilar.

1: **function GroundBisimulation**$(t, u)$ =
2: $NonBisim := \emptyset$
3: **function Bisim**$(t, u)$ = **try** {
4: $Bisim := \emptyset$
5: $Visited := \emptyset$
6: $Assumed := \emptyset$
7: **return Match**$(t, u, Visited)$
8: } **catch WrongAssumptionException** $\Rightarrow$ **Bisim**$(t, u)$
9:
10: **function Match**$(t, u, Visited)$ $\triangleright$ $t = \langle P, \rho \rangle$ *and* $u = \langle Q, \sigma \rangle$

$\quad Visited{:=}Visited \cup \{(t,u)\}$<br>
$\quad b{:=}\bigwedge_{\alpha\in \mathbf{Act(t)}} \mathbf{MatchAction}(\alpha,t,u,Visited)$<br>
$\quad \bar{b}{:=}\bigwedge_{\alpha\in \mathbf{Act(u)}} \mathbf{MatchAction}(\alpha,u,t,Visited)$<br>
$\quad b_{c_1}{:=}qv(P) = qv(Q)$<br>
$\quad b_{c_2}{:=}tr_{qv(P)}(\rho) = tr_{qv(P)}(\sigma)$<br>
$\quad b_{res}{:=}b \wedge \bar{b} \wedge b_{c_1} \wedge b_{c_2}$<br>
$\quad$ **if** $b_{res}$ is **tt then** $Bisim = Bisim \cup \{(t,u)\}$<br>
$\quad$ **else if** $b_{res}$ is **ff then**<br>
$\qquad NonBisim = NonBisim \cup \{(t,u)\}$<br>
$\qquad$ **if** $(t,u) \in Assumed$ **then**<br>
$\qquad\quad$ **raise WrongAssumptionException**<br>
$\quad$ **return** $b_{res}$<br>
<br>
**function MatchAction**$(\alpha,t,u,Visited)$<br>
$\quad$ **switch** $\alpha$ **do**<br>
$\qquad$ **case** $c!$<br>
$\qquad\quad$ **for** $t \xrightarrow{c!e_i} \Delta_i$ **do**<br>
$\qquad\qquad$ Assume $\{t_k\}_{t_k\in\lceil\Delta_i\rceil}$ and $\{u_j\}_{u \overset{c!e'_j}{\Longrightarrow} \Gamma \wedge e_i = e'_j \wedge u_j \in \lceil\Gamma\rceil}$<br>
$\qquad\qquad \mathcal{R}{:=} \{(t_k,u_j) | \mathbf{Close}(t_k,u_j,Visited) = \mathbf{tt}\}$<br>
$\qquad\qquad \theta{:=}\mathbf{LP}(\Delta_i,u,\alpha,\mathcal{R})$<br>
$\qquad\quad$ **return** $\bigwedge_i \theta_i$<br>
$\qquad$ **otherwise**<br>
$\qquad\quad$ **for** $t \xrightarrow{\alpha} \Delta_i$ **do**<br>
$\qquad\qquad$ Assume $\{t_k\}_{t_k\in\lceil\Delta_i\rceil}$ and $\{u_j\}_{u \overset{\alpha}{\Rightarrow} \Gamma \wedge u_j \in \lceil\Gamma\rceil}$<br>
$\qquad\qquad \mathcal{R}{:=} \{(t_k,u_j) | \mathbf{Close}(t_k,u_j,Visited) = \mathbf{tt}\}$<br>
$\qquad\qquad \theta{:=}\mathbf{LP}(\Delta_i,u,\alpha,\mathcal{R})$<br>
$\qquad\quad$ **return** $\bigwedge_i \theta_i$<br>
<br>
**function Close**$(t,u,Visited)$<br>
$\quad$ **if** $(t,u) \in Bisim$ **then return tt**<br>
$\quad$ **else if** $(t,u) \in NonBisim$ **then return ff**<br>
$\quad$ **else if** $(t,u) \in Visited$ **then**<br>
$\qquad Assumed = Assumed \cup \{(t,u)\}$<br>
$\qquad$ **return tt**<br>
$\quad$ **else return Match**$(t,u,Visited)$

Now let us prove the termination and correctness of the algorithm.

**Theorem 1 (Termination).** *Given two configurations t and u, the function **GroundBisimulation(t,u)** always terminates.*

*Proof.* The algorithm starts with two empty sets *NonBisim* and *Bisim*. The next action to perform is detected in **Match**. Then it invokes function **MatchAction** to find the next new pair of configurations and recursively call function

**Match** to check them. Once a state pair is checked to be non-bisimilar in function **Match**, it is added into $NonBisim$. Meanwhile, if it is also contained in the set $Assumed$, the algorithm restarts a new execution of **Bisim**. Let $k$ denote the number of executions of **Bisim**, and $NonBisim_k$ be the set $NonBisim$ at the end of $\mathbf{Bisim}_k$. It is easy to show by induction that $NonBisim_k \subset NonBisim_{k+1}$ for any $k \geq 0$. Since the system under consideration is finite-state, there always exists some $n$ such that $NonBisim_n$ is the largest set of non-bisimilar state pairs and $\mathbf{Bisim}_n$ is the last execution of **Bisim**.

After the execution of $\mathbf{Bisim}_n$, no more exceptions will be raised. Each time **Match** is executed with $t$ and $u$ as its parameters, we add $(t, u)$ into $Visited$. The quantum variables and the configurations of the quantum registers for $t$ and $u$ are compared. When no more state pairs are added into $Visited$, the function $Match$ will not be invoked again and the whole algorithm will terminate. □

**Theorem 2 (Correctness).** *Given two configurations* $t$ *and* $u$ *from two pLTSs,* $\boldsymbol{Bisim}(t, u)$ *returns true if and only if they are ground bisimilar.*

*Proof.* Let $\mathbf{Bisim}_n$ be the last execution of **Bisim**. Let $NonBisim_n$ and $Bisim_n$ be the values of the two sets $NonBisim$ and $Bisim$, respectively, recording the checked state pairs at the end of $\mathbf{Bisim}_n$. By inspecting **Match**, we know that $NonBisim_n \cap Bisim_n = \emptyset$.

Let us analyse the result returned by $\mathbf{Bisim}_n$, which is the output of the function call $\mathbf{Match}(t, u, Visited)$. If the result is $false$ then one of the conjuncts in $b_{res}$ is invalid, which means that one of the three cases discussed in the beginning of Section 3 occurs, thus $t$ and $u$ are indeed non-bisimilar. If the return is $true$ then there is $Bisim_n = Visited_n \backslash NonBisim_n$. For each pair $(t, u) \in Bisim_n$, all the conjuncts in $b_{res}$ must be true. Both $t$ and $u$ must have the same set of free quantum variables and the same density operators. In addition, they have matching transitions. That is, for any action $\alpha$, if $t \xrightarrow{\alpha} \Delta$ then there exists some weak distribution $\Theta$ such that $u \stackrel{\alpha}{\Longrightarrow} \Theta$ and $\Delta \ \mathcal{R}^\circ \ \Theta$. This is true because (i) the relation $\mathcal{R}$ in function **MatchAction** is correctly constructed, and (ii) the lifted relation $\mathcal{R}^\circ$ exists. Below we argue for (i); the existence of the lifting operation in (ii) relies on the validity of the predicate **LP** whose correctness is established by Theorem 9 in [28].

The algorithm adds a pair into $Assumed_n$ if the pair to be checked has already been visited and passed the bisimulation checking conditions. It implies that $Assumed_n \subseteq Visited_n$. Furthermore, as there is no wrong assumption detected after the execution of $\mathbf{Bisim}_n$, we have $Assumed_n \subseteq Bisim_n$ which implies that $Bisim_n = Assumed_n \cup Bisim_n$. So $Bisim_n$ constitutes a bisimulation relation containing the initial state pair $(t, u)$. □

Before concluding this section, we analyse the time complexity of the algorithm.

**Theorem 3 (Complexity).** *Let the number of configurations reachable from* $t$ *and* $u$ *be* $n$*. The time complexity of function* $\boldsymbol{Bisim}(t, u)$ *is polynomial in* $n$*.*

*Proof.* The number of state pairs is at most $n^2$. The number of state pairs examined in the $k$th execution of **Bisim** is at most $O(n^2 - k)$. Therefore, the total number of state pairs examined is as most $O(n^2+(n^2-1)+...+1) = O(n^4)$. Note that each state has finitely many outgoing transitions. Given a transition, to check if there exists a weak matching transition, we call the function **LP** at most once, the construction of a flow network and solving the linear programming problem are both polynomial in $n$ if we use the algorithm in [28]. Consequently, the whole algorithm is also polynomial in $n$. □

For the strong version of ground bisimulation, we are only concerned with the matching of strong transitions. Therefore, Algorithm 1 can be simplified and there is no need of the predicate **LP** in the function **MatchAction**.

## 4 Implementation and Experiments

In this section, we report on an implementation of our approach and provide the experimental results of verifying several quantum communication protocols.

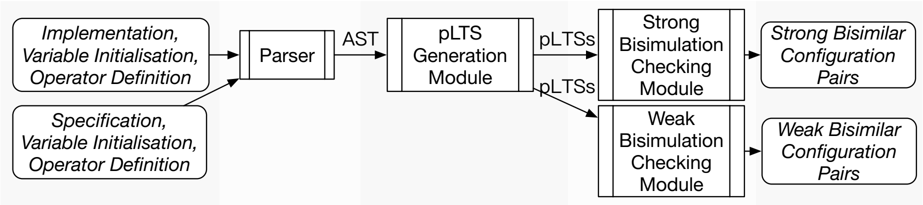

**Fig. 3.** Verification workflow.

### 4.1 Implementation

We have implemented both strong and weak ground bisimulation checkers in Python 3.7. The workflow of our tool is sketched in Figure 3. The tool consists of a pLTS generation module and two bisimulation checking modules, devoted to modeling and verification, respectively. The input of this tool is a specification and an implementation of a quantum protocol, both described as qCCS processes, the definition of user-defined operators, as well as an initialisation of classical and quantum variables. Unlike classical variables, the initialisation of all quantum variables, deemed as a quantum register, is accomplished at the same time so to allow for superposition states. The final output of the tool is a result indicating whether the specification and the implementation are bisimilar under the same initial states. The algorithm also stores the bisimilar state pairs and non-bisimilar state pairs in two tables.

The pLTS generation module acts as a preprocessing unit before the verification task. It first translates the input qCCS processes into two abstract syntax trees (ASTs) by a parser. Then the ASTs are transformed into two pLTSs according to the operational semantics given in Figure 2, using the user-defined operators and the initial values of variables. The weak bisimulation checking module implements the weak ground bisimilarity checking algorithm we defined in the last section. It checks whether the initial states of the two generated pLTSs are weakly bisimilar.

The tool is available in [24], where we also provide all the examples for the experiments to be discussed in Section 4.3.

#### 4.2 BB84 Quantum Key Distribution Protocol

To illustrate the use of our tool, we formalise the BB84 quantum key distribution protocol. Our formalisation follows [11], where a manual analysis of the protocol is provided. Now we perform automatic verification via the ground bisimulation checker.

The BB84 protocol provides a provably secure way to create a private key between two partners with a classical authenticated channel and a quantum insecure channel between them. The protocol does not make use of entangled states. It ensures its security through the basic property of quantum mechanics: if the states to be distinguished are not orthogonal, such as $|0\rangle$ and $|+\rangle$, then information gain about a quantum state is only possible at the expense of changing the state. Let the sender and the receiver be *Alice* and *Bob*, respectively. The basic BB84 protocol with a sequence of qubits $\tilde{q}$ with size $n$ goes as follows:

1. *Alice* randomly generates two sequences of bits $\tilde{B}_a$ and $\tilde{K}_a$ using her qubits $\tilde{q}$. Note that $\tilde{q}$ here are auxiliary qubits which are not modified in this step.
2. *Alice* sets the state of $\tilde{q}$, such that the $i$th bits of $\tilde{q}$ is $|x_y\rangle$ where $x$ and $y$ are the $i$th bits of $\tilde{B}_a$ and $\tilde{K}_a$, and respectively, $|0_0\rangle = |0\rangle$, $|0_1\rangle = |1\rangle$, $|1_0\rangle = |+\rangle = (|0\rangle + |1\rangle)/\sqrt{2}$ and $|1_1\rangle = |-\rangle = (|0\rangle - |1\rangle)/\sqrt{2}$.
3. *Alice* sends her qubits $\tilde{q}$ to *Bob*.
4. *Bob* randomly generates a sequence of bits $\tilde{B}_b$ using his qubits $\tilde{q}'$.
5. *Bob* measures the $i$th qubit of $\tilde{q}$ he received from *Alice* according to the basis determined by the $i$th bit of $\tilde{B}_b$. Respectively, the basis is $\{|0\rangle, |1\rangle\}$ if it is 0 and $\{|+\rangle, |-\rangle\}$ if it is 1.
6. *Bob* sends his choice of measurements $\tilde{B}_b$ to *Alice*, and after receiving the information, *Alice* sends her $\tilde{B}_a$ to *Bob*.
7. *Alice* and *Bob* match two sequences of bits $\tilde{B}_a$ and $\tilde{B}_b$ to determine at which positions the bits are equal. If the bits match, they keep the corresponding bits of $\tilde{K}_a$ and $\tilde{K}_b$. Otherwise, they discard them.

After the execution of the basic BB84 protocol, the remaining bits of $\tilde{K}_a$ and $\tilde{K}_b$ should be the same, provided that the communication channels are perfect and there is no eavesdropper.

*Implementation.* For simplicity, we assume that the sequence $\tilde{q}$ consists of only one qubit. This is enough to reflect the essence of the protocol. The other qubits used below are auxiliary qubits for the operation *Ran*.

$$Alice \stackrel{def}{=} Ran[q_1; B_a].Ran[q_1; K_a].Set_{K_a}[q_1].H_{B_a}[q_1].\underline{A2B}!q_1. \\ b2a?B_b.a2b!B_a.key_a!cmp(K_a, B_a, B_b).\mathbf{nil};$$

$$Bob \stackrel{def}{=} \underline{A2B}?q_1.Ran[q_2; B_b].M_{B_b}[q_1; K_b].b2a!B_b. \\ a2b?B_a.key_b!cmp(K_b, B_a, B_b).\mathbf{nil};$$

$$BB84 \stackrel{def}{=} (Alice||Bob) \setminus \{a2b, b2a, \underline{A2B}\}$$

where there are several special operations:

- $Ran[q; x] = Set_+[q].M_{0,1}[q; x].Set_0[q]$, where $Set_+$ (resp.$Set_0$) is the operation which sets a qubit it applies on to $|+\rangle$ (resp.$|0\rangle$), $M_{0,1}[q; x]$ is the quantum measurement on $q$ according to the basis $\{|0\rangle, |1\rangle\}$ and stores the result into $x$.
- $Set_K[q]$ sets the qubit $q$ to the state $|K\rangle$.
- $H_B[q]$ applies $H$ or does nothing on the qubit $q$ depending on whether the value of $B$ is 1 or 0.
- $M_B[q; K]$ is the quantum measurement on $q$ according to the basis $\{|+\rangle, |-\rangle\}$ or $\{|0\rangle, |1\rangle\}$ depending on whether the value of $B$ is 1 or 0.
- $cmp(x, y, z)$ returns $x$ if $y$ and $z$ match, and $\epsilon$, meaning it is empty, if they do not match.

*Specification.* The specification can be defined as follows using the same operations:

$$BB84_{spec} \stackrel{def}{=} Ran[q_1; B_a].Ran[q_1; K_a].Ran[q_2; B_b] \\ .(key_a!cmp(K_a, B_a, B_b).\mathbf{nil}||key_b!cmp(K_a, B_a, B_b).\mathbf{nil}).$$

*Input.* For the implementation of $BB84$, we need to declare the following variables and operators in the input attached to it.

- The classical bits are named $B_a$, $K_a$ for *Alice* and $B_b$, $K_b$ for *Bob*.
- The qubits are declared together as a vector $|q_1, q_2\rangle$. The vector always needs an initial value. We can set it to be $|00\rangle$ in this example.

When modelling the protocol, we use several operators. They should be defined and their definitions are part of the input.

- The operator *Ran* involves two operators $Set_+$, $Set_0$ and a measurement $M_{0,1}$ measuring the qubit according to the basis $\{|0\rangle, |1\rangle\}$.
- $Set_K$ needs $Set_0$ and $Set_1$.
- $H_B$ requires the Hadamard gate $H$.
- $M_B$ uses the measurement $M_{+,-}$ which measures the qubit according to the basis $\{|+\rangle, |-\rangle\}$.

The function *cmp* is treated as an in-built function, so there is no need to define it in the input.

For the specification $BB84_{spec}$, we only declare the classical bits $B_a$, $B_b$, $K_a$, qubits $q_1$, $q_2$ and the operator *Ran*. The variables and operators declared here are the same as those in the input of the implementation.

*Output.* Taking the input discussed above, the tool first generates two pLTSs, with over 150 states for the implementation and 80 states for the specification, and then runs the ground bisimulation checking algorithm. As we can see from the fifth row in Table 1, our tool confirms that $\langle BB84, \rho_0\rangle \approx \langle BB84_{spec}, \rho_0\rangle$, where $\rho_0$ denotes the initial state of the quantum register, thus the implementation is faithful to the specification. In the output of the tool, there is an enumeration of 1084 pairs of non-bisimilar states and 3216 pairs of bisimilar states. The pLTSs and the state pairs can be found in [24].

#### 4.3 Experimental Results

We conducted experiments on several quantum communication protocols with a few different input variables. Table 1 provides a summary of our experimental results obtained on a macOS machine with an Intel Core i7 2.5 GHz processor and 16GB of RAM.

| **Weak ground bisimulation** | | | | | | | |
|---|---|---|---|---|---|---|---|
| **Program** | **Variables** | **Bisi** | **Impl** | **Spec** | **N** | **B** | **ms** |
| Super-dense coding | $q_1q_2 = \|00\rangle$, $x = 1$ | Yes | 16 | 5 | 9 | 20 | 259 |
| | $q_1q_2 = \|00\rangle$, $x = 5$ | No | 6 | 2 | - | - | 2 |
| Super-dense coding (modified) | $q_1q_2 = \|00\rangle$, $x = 5$ | Yes | 8 | 5 | 5 | 12 | 110 |
| Teleportation | $q_1q_2q_3 = \|100\rangle$ | Yes | 34 | 3 | 22 | 22 | 232 |
| | $q_1q_2q_3 = \frac{1}{\sqrt{2}}\|000\rangle+\frac{1}{\sqrt{2}}\|100\rangle$ | Yes | 34 | 3 | 22 | 22 | 264 |
| | $q_1q_2q_3 = \frac{\sqrt{3}}{2}\|000\rangle+\frac{1}{2}\|100\rangle$ | Yes | 34 | 3 | 22 | 22 | 239 |
| Secret Sharing | $q_1q_2q_3q_4 = \|1000\rangle$ | Yes | 103 | 3 | 65 | 65 | 1339 |
| | $q_1q_2q_3q_4=\frac{1}{\sqrt{2}}\|0000\rangle+\frac{1}{\sqrt{2}}\|1000\rangle$ | Yes | 103 | 3 | 65 | 65 | 1252 |
| | $q_1q_2q_3q_4=\frac{\sqrt{3}}{2}\|0000\rangle+\frac{1}{2}\|1000\rangle$ | Yes | 103 | 3 | 65 | 65 | 1187 |
| BB84 | $q_1q_2 = \|00\rangle$ | Yes | 152 | 80 | 1084 | 3216 | 130163 |
| BB84 (with eavesdropper) | $q_1q_2q_3 = \|000\rangle$ | Yes | 1180 | 352 | 121072 | 75392 | 55728587 |
| B92 | $q_1q_2 = \|00\rangle$ | Yes | 64 | 80 | 466 | 1284 | 34522 |
| E91 | $q_1q_2q_3q_4 = \|0000\rangle$ | Yes | 124 | 80 | 964 | 2676 | 113840 |

**Table 1.** Experimental results. The columns headed by **Impl** and **Spec** show the numbers of nodes contained in the generated pLTSs of the implementations and specifications, respectively. Column **N** shows the sizes of the sets of non-bisimilar state pairs and Column **B** shows the sizes of the sets of bisimilar state pairs. Column **ms** shows the time cost of the verification in milliseconds.

In each case, we report the final outcome (whether an implementation is ground bisimilar to its specification), the number of nodes in two pLTSs, the numbers of non-bisimilar and bisimilar state pairs in *NonBisim* and *Bisim*, respectively, as well as the verification time of our ground bisimulation checking algorithm. The time cost excludes the part of pLTS generation which takes around one second in all the examples.

Besides the protocol discussed in Section 4.2, we also verify other ones that make use of entangled qubits such as the teleportation and the quantum secrect sharing protocol. For quantum key distribution protocols, we conduct experiments on the BB84, the B92 and the E91.

Not all the cases in Table 1 give the size of the set $NonBisim$ of non-bisimilar state pairs, as the bisimulation checking algorithm may immediately terminate once a negative verification result is obtained, i.e. the two initial states are not bisimilar.

**Data Availability Statement**
The datasets generated and/or analyzed during the current study are available in the figshare repository: https://doi.org/10.6084/m9.figshare.11874942.v1.

### 5 Conclusion and Future Work

We have presented an on-the-fly algorithm to check ground bisimulation for quantum processes in qCCS, and a simpler algorithm for strong ground bisimulation. Based on the algorithms, we have developed a tool to verify quantum communication protocols modelled as qCCS processes. We have carried out experiments on several non-trivial quantum communication protocols from superdense coding to key distribution and found the tool helpful.

As to future work, several interesting problems remain to be addressed. For example, a limitation of the current work is to compare quantum processes with predetermined states of quantum registers. Indeed, there are occasions where one would expect two processes to be equivalent for arbitrary initial states. It is infeasible to enumerate all those states. Then the symbolic bisimulations proposed in [11] will be useful. We are considering to implement the algorithm for symbolic ground bisimulation, and then tackle the more challenging symbolic open bisimulation, both proposed in that work. Another problem occurs in the experiment of Section 4.2. The example tested one qubit instead of a sequence of qubits because more qubits lead to a drastic growth of the running time, which shows a limitation of the current approach of explicitly representing state spaces.

### Appendix

Algorithm 1 needs to check the condition that if $t \xrightarrow{\alpha} \Delta$ then there exists some $\Theta$ such that $u \stackrel{\hat{\alpha}}{\Longrightarrow} \Theta$ and $\Delta \ \mathcal{R}^{\circ} \ \Theta$. We use as a subroutine a procedure of [28] to reduce the problem to a network flow problem that can be solved in polynomial time.

Technically, we construct a network graph $G(\Delta, u, \alpha, \mathcal{R}) = (V, E)$ defined as follows. Let $S$ be the set of reachable states, and $\mathcal{R}$ be a binary relation on the states.

Let $\vartriangle$ and $\blacktriangledown$ be two vertices that represent the source and the sink of the network, respectively. For each visible action $\alpha$, the set of vertices $V$ is given below

$$V = \{\vartriangle, \blacktriangledown\} \cup S \cup S^{tr} \cup S_\alpha \cup S_\alpha^{tr} \cup S_\perp \cup S_\mathcal{R}$$

where

$$\begin{aligned}
S^{tr} &= \{v^{tr} | tr = v \xrightarrow{\beta} \Gamma,\ \beta \in \{\alpha, \tau\}\};\\
S_\alpha &= \{v_\alpha | v \in S\};\\
S_\alpha^{tr} &= \{v_\alpha^{tr} | v^{tr} \in S^{tr}\};\\
S_\perp &= \{v_\perp | v \in S\};\\
S_\mathcal{R} &= \{v_\mathcal{R} | v \in S\}.
\end{aligned}$$

and the set of edges $E$ is

$$E = \{(\vartriangle, u)\} \cup L_1 \cup L_\alpha \cup L_2 \cup L_\perp^\alpha \cup L_\mathcal{R}$$

where

$$\begin{aligned}
L_1 &= \{(v, v^{tr}), (v^{tr}, v') | tr = v \xrightarrow{\tau} \Gamma,\ v' \in \lceil \Gamma \rceil\};\\
L_\alpha &= \{(v, v_\alpha^{tr}), (v_\alpha^{tr}, v'_\alpha) | tr = v \xrightarrow{\alpha} \Gamma,\ v'_\alpha \in \lceil \Gamma \rceil\};\\
L_2 &= \{(v_\alpha, v_\alpha^{tr}), (v_\alpha^{tr}, v'_\alpha) | tr = v_\alpha \xrightarrow{\tau} \Gamma,\ v'_\alpha \in \lceil \Gamma \rceil\};\\
L_\perp^\alpha &= \{(u_\alpha, u_\perp) | u \in S\};\\
L_R &= \{(s_\perp, s'_\mathcal{R}), (s'_\mathcal{R}, \blacktriangledown) | (s, s') \in \mathcal{R}\}.
\end{aligned}$$

For the invisible action $\tau$, the definition is similar: $V = \{\vartriangle, \blacktriangledown\} \cup S \cup S^{tr} \cup S_\perp \cup S_\mathcal{R}$ and $E = \{(\vartriangle, u)\} \cup L_1 \cup L_\perp \cup L_\mathcal{R}$ where $L_\perp = \{(s, s_\perp) \mid s \in S\}$.

If $\alpha$ is a visible action, we consider the following linear programming problem associated to $G(\Delta, u, \alpha, \mathcal{R})$:

$$\max \sum\nolimits_{(s,v) \in E} -f_{s,v}$$

subject to

$$\begin{aligned}
&f_{s,v} \geq 0 && \text{for each } (s, v) \in E\\
&f_{\vartriangle,u} = 1 &&\\
&f_{v_\mathcal{R},\blacktriangledown} = \Delta(v) && \text{for each } v \in S\\
&\sum\nolimits_{(s,v) \in E} f_{s,v} - \sum\nolimits_{(v,w) \in E} f_{v,w} = 0 && \text{for each } v \in V \setminus \{\vartriangle, \blacktriangledown\}\\
&f_{v^{tr},v'} - \Gamma(v') \cdot f_{v,v^{tr}} = 0 && \text{for each } tr = v \xrightarrow{\tau} \Gamma \text{ and } v' \in \lceil \Gamma \rceil\\
&f_{v_\alpha^{tr},v'_\alpha} - \Gamma(v') \cdot f_{v,v_\alpha^{tr}} = 0 && \text{for each } tr = v \xrightarrow{\alpha} \Gamma \text{ and } v' \in \lceil \Gamma \rceil\\
&f_{v_\alpha^{tr},v'_\alpha} - \Gamma(v') \cdot f_{v_\alpha,v_\alpha^{tr}} = 0 && \text{for each } tr = v \xrightarrow{\tau} \Gamma \text{ and } v' \in \lceil \Gamma \rceil
\end{aligned}$$

Note that the fourth constraint is referred to as the flow-conservation constraints. The last three constraints link the source state and the result distribution.

For the invisible action $\tau$, the linear programming problem associated to the network $G(\Delta, u, \tau, \mathcal{R})$ is the same as above except that the last two constraints are dropped.

We denote by $\mathbf{LP}(\Delta, u, \alpha, \mathcal{R})$ the predicate that is true if and only if the linear programming problem above has a solution.

## References

[1] Aaronson, S., Gottesman, D.: Improved simulation of stabilizer circuits.Physical Review A **70**(052328) (2004)

[2] Abramsky, S., Coecke, B.: A categorical semantics of quantum protocols. In: Proceedings of the 19th IEEE Symposium on Logic in Computer Science. pp. 415–425. IEEE Computer Society (2004)

[3] Ardeshir-Larijani, E., Gay, S.J., Nagarajan, R.: Automated equivalence checking of concurrent quantum systems. ACM Transactions on Computational Logic 19(4), 28:1–28:32 (2018)

[4] Baier C, Engelen B, Majster-Cederbaum ME (2000). Deciding bisimilarity and similarity for probabilistic processes. Journal of Computer and System Sciences.

[5] Bennett, C.H., Brassard, G.: Quantum cryptography: Public-key distribution and coin tossing. In: Proceedings of the IEEE International Conference on Computer, Systems and Signal Processing. pp. 175–179 (1984)

[6] Davidson, T.A.S.: Formal Verification Techniques using Quantum Process Calculus. Ph.D. thesis, University of Warwick (2011)

[7] Deng, Y.: Semantics of Probabilistic Processes: An Operational Approach.Springer (2015)

[8] Deng, Y., Du, W.: A local algorithm for checking probabilistic bisimilarity. In: Proceedings of the 4th International Conference on Frontier of Computer Science and Technology. pp. 401–407. IEEE Computer Society (2009)

[9] Deng Yuxin, Feng Yuan, Baeten Jos C M, Ball Tom, de Boer Frank S (2012). Open Bisimulation for Quantum Processes. Theoretical Computer Science.

[10] Feng Y, Duan R, Ji Z, Ying M (2007). Probabilistic bisimulations for quantum processes. Information and Computation.

[11] Feng Y, Deng Y, Ying M (2020). Symbolic bisimulation for quantum processes. ACM Transactions on Computational Logic.

[12] Feng, Y., Duan, R., Ying, M.: Bisimulation for quantum processes. In: Proceedings of the 38th Annual ACM SIGPLAN-SIGACT Symposium on Principles of Programming Languages. pp. 523–534. ACM (2011)

[13] Feng Yuan, Hahn Ernst Moritz, Turrini Andrea, Zhang Lijun, Bjørner Nikolaj, de Boer Frank (2015). QPMC: A Model Checker for Quantum Programs and Protocols. FM 2015: Formal Methods.

[14] Fernandez, J.C., Mounier, L.: Verifying bisimulations "on the fly". In: Proceedings of the 3rd International Conference on Formal Description Techniques for Distributed Systems and Communication Protocols. pp. 95–110. North-Holland (1990)

[15] Gay, S.J., Nagarajan, R.: Communicating quantum processes. In: Palsberg, J., Abadi, M. (eds.) Proceedings of the 32Nd ACM SIGPLAN-SIGACT Symposium on Principles of Programming Languages. pp. 145–157 (2005)

[16] Gay Simon J, Nagarajan Rajagopal, Papanikolaou Nikolaos, Gupta Aarti, Malik Sharad (2008). QMC: A Model Checker for Quantum Systems. Computer Aided Verification.

[17] Hennessy M, Lin H (1995). Symbolic bisimulations. Theoretical Computer Science.

[18] Jorrand, P., Lalire, M.: Toward a quantum process algebra. In: Proceedings of the 1st Conference on Computing Frontiers. pp. 111–119. ACM (2004)

[19] Kissinger, A.: Pictures of Processes: Automated Graph Rewriting for Monoidal Categories and Applications to Quantum Computing. Ph.D. thesis, University of Oxford (2011)

[20] Kubota T, Kakutani Y, Kato G, Kawano Y, Sakurada H (2016). Semi-automated verification of security proofs of quantum cryptographic protocols. Journal of Symbolic Computation.

[21] Lalire M (2006). Relations among quantum processes: Bisimilarity and congruence. Mathematical Structures in Computer Science.

[22] Liu, T., Li, Y., Wang, S., Ying, M., Zhan, N.: A theorem prover for quantum hoare logic and its applications. CoRR abs/1601.03835 (2016)

[23] Milner, R.: Communication and Concurrency. Prentice-Hall (1989)

[24] Qin, X., Deng, Y., Du, W.: QBisim (2020), https://github.com/MartianQXD/QBisim

[25] Sangiorgi D (1996). A theory of bisimulation for the pi-calculus. Acta Informatica.

[26] Selinger P (2004). Towards a quantum programming language. Mathematical Structures in Computer Science.

[27] Shor P, Preskill J (2000). Simple proof of security of the BB84 quantum key distribution protocol. Physical Review Letters.

[28] Turrini A, Hermanns H (2015). Polynomial time decision algorithms for probabilistic automata. Information and Computation.

[29] Ying, M., Feng, Y., Duan, R., Ji, Z.: An algebra of quantum processes. ACMTransactions on Computational Logic **10**(3), 1–36 (2009)

[30] Ying, M.: Foundations of Quantum Programming. Morgan Kaufmann (2016)

